# When Parent–Teacher Collaboration Turns Violent: Corporal Punishment in American Schools and Subsequent (Secondary) Trauma

**DOI:** 10.3390/children12060684

**Published:** 2025-05-26

**Authors:** Da’Shay Templeton, Ruslan Korchagin, Bree Valla, Jesse R. Ford

**Affiliations:** 1 Educational Leadership, California Lutheran University, Thousand Oaks, CA 61360, USA; rkorchag@callutheran.edu (R.K.); bvalla@callutheran.edu (B.V.); 2 Teacher Education and Higher Education, University of North Carolina at Greensboro, Greensboro, NC 27412, USA; jrfordjr@uncg.edu

**Keywords:** corporal punishment, trauma, trauma-informed policy, childhood trauma theory

## Abstract

**Methods.** Through the lens of childhood trauma theory, a qualitative phenomenological study was conducted using purposive and snowball sampling methods to gain a deeper understanding of the experiences of former students with corporal punishment and how those experiences have shaped their academic and psychological outcomes. Interviews were conducted via Zoom with 19 men and women of different ages and races who attended schools in Mississippi. **Results/Conclusions.** The study revealed that parents and school personnel collaborated to punish the student corporally both on campus and at home. Related, beaten students did not share their punishment with their parents/caregivers, and if their families did find out, they received another beating at home. There was a general lack of consistency in how and who administered corporal punishment. In addition to the well-documented ways that corporal punishment is administered in school, we also found that students were made to hold painful positions or perform painful tasks. There were also peer effects of trauma, with students experiencing fear or anger following a friend or classmate being beaten in front of them. Race was an influence if the abused students felt that their punishment was racist, with Black American participants feeling there were racial undertones regardless of the perpetrator’s race. The study’s findings align with those of previously conducted research, but also extend them and can be used to create policy to allow schools to address trauma and create instructional practices that eliminate the fear and racial disparities that have been proven to exist in schools with corporal punishment.

## 1. When Parent–Teacher Collaboration Turns Violent: Corporal Punishment in American Schools and Subsequent (Secondary) Trauma

When does collaboration between teachers and parents turn violent? When does someone else’s trauma become your own? In several states, corporal punishment in the home results in a visit from Child Protection Services and, in some cases, incarceration; yet, select school personnel are exempt from child abuse charges for corporal punishment in school (CPS) [1]. In the U.S., twenty-three states do not expressly prohibit the use of CPS, making it a form of state-sanctioned violence [2]. School personnel use brutal tactics and techniques to dole out CPS via hands, feet, or objects such as canes, rulers, paddles, yardsticks, and belts. The assault takes various forms: choking, kicking, punching, pinching, slapping, and shaking as well as requiring the child to hold painful positions for extended periods of time [3,4].

CPS has deleterious consequences for the youth on whom the pain is inflicted; however, the extent to which these consequences extend to peer effects has yet to be explored in the literature and is investigated in-depth in this study. CPS is associated with negative life outcomes, including mental (e.g., increased aggressive behaviors, maladaptive behaviors, depression, and anxiety) [5,6], physical (increased bodily injury) [1], academic (increased dropout rates and chronic absenteeism and decreased GPA) [7,8], and socio-emotional effects (decreased sense of belonging and trust in authority figures) [9].

While the legality and administration of CPS have declined in America, about 600 students are still struck each day [1]. Instances of CPS are often underreported, so these estimates could be greater [5]. Whether a child is beaten at school is random, but in Mississippi—a state with the highest rates of CPS in America—the probability is increased [1], making it an ideal location for a case study. Using in-depth interviews of 19 former Mississippi public school students, we sought to answer this question: How has CPS shaped the academic and psychological outcomes of Mississippi public school students? This study contributes to a long line of research studies on CPS but provides an in-depth and contemporary analysis of a state with the highest instances of corporal punishment in schools, particularly regarding how parents and teachers work together to administer corporal punishment as well as the secondary effects of trauma on peers.

We found that students experienced primary, compounded, and secondary trauma as well as secrecy and shame for fear of telling their guardians and receiving an additional corporal punishment. Generally, there were a perceived lack of consistency and increased racial biases. Lastly, we recorded their recommendations for change, which align well with previous research. The findings underscore the critical role school personnel and parents play in creating traumatic conditions for vulnerable youth and their peers. The study concludes with practical suggestions that the state, parents, and school personnel can implement to protect our students and increase their success. The following section outlines the theory used to anchor and guide this study.

## 2. Childhood Trauma Theory

In order to investigate the long-term effects of CPS on Mississippi public school students, this study leverages childhood trauma theory. Childhood trauma theory has no single founder; instead, it has been developed over time by scholars in the fields of psychology, psychiatry, and psychoanalysis. Traumatic events are experienced by many children, and their psychological and developmental consequences are often severe [10]. The term childhood trauma refers to the harm, potential harm, or threat of harm caused by the actions of a child’s caregiver [11]. As defined by the American Psychiatric Association, traumatic exposure occurs when an individual is confronted with death, serious injury, or other threats to physical integrity [12]. Men and women report physical abuse, physical neglect, and emotional abuse as the most common forms of trauma [13]. The effects of interpersonal trauma often manifest in a greater number of global and profound changes in children than they do in adults, who are conceivably more resistant to stress and possess more cognitive resources to mitigate risks and promote resilience. Children who experience such events are likely to lose their sense of self-esteem, their sense of lovability, their sense of vulnerability, and their faith in family, friends, and a higher power as a result [9].

In children, posttraumatic stress and its pathological manifestation, posttraumatic stress disorder, have been the most extensively studied psychological consequences of traumatic exposure [12]. As a result of prolonged exposure to trauma, changes in brain structure and function may also occur, particularly in areas associated with emotional regulation and fear processing [13]. In accordance with the findings of neuroscientific researchers, trauma can adversely affect a child’s academic health in a number of ways, including impaired brain development related to language and communication, impaired self-esteem, impaired learning, compromised ability to pay attention in class, diminished memory, difficulty following instructions, poor organizational skills, and difficulty grasping cause-and-effect relationships. Researchers have demonstrated that protective and compensatory experiences are critical to the healing and improvement of those who have suffered adverse childhood experiences [14,15,16,17,18]. The sooner these protective measures are implemented, the sooner the vicious cycle that leads to failure or social and personal disadvantage will end. The next section reviews the extant literature.

## 3. Literature Review

### Secondary Trauma

We first review the literature on secondary trauma, which we propose researchers leverage in addition to childhood trauma theory. Hearing about or witnessing another person’s traumatic experiences may cause secondary trauma, also known as secondary traumatic stress, vicarious traumatization, or compassion fatigue [19]. Figley [20] conceptualizes secondary traumatic stress as the natural, subsequent feelings and behaviors associated with knowing about a traumatizing event experienced by a significant other as well as the stress caused by helping or wanting to assist someone in distress. People who witness traumatic events or care for trauma survivors suffer secondary trauma. It occurs when a person is exposed to a traumatic event experienced by another person [21]. Experiencing trauma situations and identifying with those in them can also cause secondary trauma reactions, especially when these experiences evoke fear reactions in the witness [22,23].

As a result of secondary traumatic stress, individuals may experience cognitive difficulties, such as intrusive thoughts and difficulty concentrating, as well as physical changes, such as insomnia or fatigue [24]. A secondary traumatic stress disorder is characterized by intrusive imagery, avoidant reactions, physiological arousal, distressing emotions, and functional impairment [25]. As a result of secondary trauma, a variety of symptoms may appear, including anger, anxiety, depression, low self-esteem, emotional exhaustion, difficulty concentrating, body aches, sleep problems, changes in eating habits, startle reactions, and an increase in addictive behaviors, as well as withdrawal from others [26].

Typically, secondary trauma of childhood refers to negative psychological experiences caused by a child’s close relationship with a traumatized individual. An emotional bond may exist between a child and a parent, guardian, relative, or anyone else with whom the child has a close relationship [26]. A child’s secondary trauma can have a significant impact on their cognitive ability, emotional health, behavior, physical health, and personal relationships [27]. In order to heal secondary trauma, children should be provided with a secure nonthreatening environment where safety and the establishment of trust are prioritized [26]. A relatively limited amount of research has been conducted on the measurement of secondary trauma, and the majority of the assessment devices that exist are designed for therapists and not for the general public or for children [26]. There are relatively few empirical studies on secondary trauma in children, and systematic, controlled research is lacking. Secondary trauma is a field that is in its infancy and relatively lacking in empirical studies.

## 4. Impacts of Corporal Punishment on Students

Corporal punishment uses a punishing stimulus after a student has exhibited an undesirable behavior with the intent that the negative experience will deter the child from repeating the offensive behavior [4,7,28]—though research supporting that notion is limited and controversial [1]. Corporal punishment is often administered using an object to inflict pain, such as a paddle or cane, which routinely leads to physical injury to the student, ranging from bruises and cuts to broken bones [4]. School personnel administer corporal punishment; they are the individuals hurting the child. The same individuals who are responsible for motivating children on campus are physically injuring them. This dichotomy is difficult for students to understand and conflicts with their sense of self-efficacy and safety in schools.

The act of hitting a child does not teach nor explain why their behavior was offensive, thereby not providing a learning opportunity for the student. For punishment to be effective in changing behavior, it must be administered immediately, consistently, and after every instance of the behavior [4]. When students cannot understand the “why” behind their punishment, coupled with the fact that it is violent punishment, their self-regulation and self-efficacy are negatively impacted. They cannot make sense of the reason for the violent punishment being inflicted on them by a school official who is supposed to protect them [1].

Students who experience corporal punishment are more likely to be depressed, suffer from anxiety, and report a disconnect from their schools [4,6], symptoms that are commonly seen in those who cannot exercise self-efficacy, resulting in an inability to recover from the violent, traumatic event [29]. Not surprisingly, students who were the victims of corporal punishment also had lower grade-point averages [4], higher dropout rates, and increased absenteeism [30,31,32,33]. Once again, these students cannot self-regulate and struggle to see the likelihood of attaining their future goals [29] because of the traumatic effect of corporal punishment. Students in southern states and Black American students have a higher chance of receiving CPS [1,34]; unfortunately, those same groups are disproportionally punished corporally at home.

## 5. Corporal Punishment in Black American Culture

As revealed in the study conducted by Patton et al. [35], Black American children are more likely to be injured or killed by their parents than by police because of white supremacy. White supremacy ensures that Black American parents and guardians beat their children to ensure that they are prepared for the expected social violence. Black American parents and guardians believe that corporal punishment is necessary to prevent the incarceration of their youth and prepare them for police violence. Patton et al. [35] argue that physical punishment is the legacy of slavery. This notion is supported by research that links slavery and lynching to increased CPS [34].

A qualitative study conducted by LeCuyer et al. [36] with southern Black American mothers of young children found that corporal punishment was considered normal in this population. Taylor et al. [37] found that Black Americans living in southern states believe that corporal punishment is a necessary component of effective parenting. Black American families use corporal punishment at higher rates than non-Latino White or Latino families, even after controlling for socioeconomic status [38]. Compared to other racial groups, Black American parents approve of corporal punishment at a higher rate [39]. At the same time, Klevens et al. [40] found that the majority of parents, regardless of their racial or ethnic affiliation, believe that most other parents hit their children.

A recent study conducted by Duong et al. [41] found some trends in the usage of corporal punishment by Black American parents. First, the change in the current social environment does not support child physical discipline, and it no longer works in favor of Black American parents. Second, as a result of feeling misunderstood and unfairly evaluated by society, Black American parents resisted social pressures not to use corporal punishment. Third, Black American parents believe corporal punishment is an effective and normal disciplinary method that worked well in their youth. Fourth, Black American parents believe that non-physical discipline leads to undesirable consequences.

## 6. Consistency in the Administration of School Discipline

For discipline to be effective, it must also be consistent [4]; however, research evidences that school discipline is inconsistent. Welsh [42] found that a substantial amount of language used in school-level training materials reflects interventionist disciplinary philosophies, such as “behavior cannot be shaped unless interventions are implemented consistently”. Irby and Clough [43] emphasized that consistency is a guiding principal of school discipline culture. They identified three ways in which consistency functions across schools. First, consistency serves as a means of maintaining collegial relations. Second, teachers employ consistency to promote fairness and equality in the treatment of their students. Third, consistency is essential to ensuring that students are aware of rules and expectations at school and can comply with them. Furthermore, Williams III [44] found that Black American students are less likely to be suspended when school administrators are more consistent.

At the same time, a group of researchers highlight issues with consistency in the implementation of school discipline. Griffith and Tyner [45] discovered that school discipline protocols are inconsistently implemented. Smith and Hains [46] highlight that school discipline policies vary depending on the philosophies and beliefs of educators guiding disciplinary practices. Kennedy et al. [47] highlight a tension between consistency and individualization in school administrators’ decisions to follow centralized school discipline guidelines. On the one hand, guidelines provide easy choices when applying school discipline. On the other hand, many administrators did not feel comfortable with the rigidity of the code and made compromises in their decisions based on numerous factors. Shabazian [48] examined how administrative perspectives shape the implementation of school discipline policy and determined that an administrator’s decision to exclude students was influenced by five normative values: productive efficiency, equality versus equity, legal liability, prescribing a cultural deficit ideology, and the notion of strict surveillance. Discipline varies by state, districts, and even schools within the same district, but below, we provide information on the legality and administration of discipline in Mississippi public schools.

## 7. Administration of School Discipline in Mississippi

Mississippi is one of the states that allows corporal punishment for students as long as they do not have disabilities [49]. According to Education Code §49001 (a), corporal punishment is the “willful infliction of, or willfully causing the infliction of, physical pain on a pupil”. Mississippi Code 37-11-57 (2) defines corporal punishment as “reasonable use of physical force or physical contact by a teacher, assistant teacher, principal or assistant principal, as may be necessary to maintain discipline, to enforce a school rule, for self-protection, or for the protection of other students from disruptive students”. Furthermore, MI Code 37-11-52 (2) states that when a “public school teacher, assistant teacher, principal or assistant principal” administers corporal punishment in a “reasonable manner” or uses “any reasonable action to maintain control and discipline of a student”, they cannot be found negligent or guilty of child abuse. In addition, they are shielded from any civil damages that could arise from a student who may suffer due to the application of corporal punishment.

Mississippi law explicitly gives the power to suspend or expel a student to the superintendent or school principal anytime their presence in the classroom disrupts the educational environment or if their presence is detrimental to the other students and the teacher (§37-9-71). If the suspension is more than 10 days, the student has the right to due process (§37-9-71). However, if a student receives corporal punishment, the student has no opportunity for due process. Based on the Mississippi Education Code and the restraints on suspension and expulsion, these forms of discipline are considered more extreme than corporal punishment. According to Mississippi governmental officials, physical infliction of pain is less injurious than having a student not attend school.

According to federal data, in 2017–2018, the most recent year with data, Mississippi had the highest rates of corporal punishment administration of any state, with nearly 30% of all incidents occurring in Mississippi [50]. In 2019, Mississippi restricted corporal punishment so that it could not be used on students with disabilities, which resulted in a decrease of over 23,000 instances of corporal punishment [51]. In 2019, Mississippi realized that the deleterious effects of corporal punishment were too extreme and inappropriate for students with disabilities. However, non-disabled students still receive corporal punishment.

School discipline, specifically corporal punishment, is applied inequitably. In 2020–2021, Mississippi students were 47.72% Black or African American, 43.13% White, 4.39% Hispanic or Latin*, 3.33% two or more races, 1.15% Asian, 0.22% American Indian or Alaskan Native, and 0.06% Native Hawaiian or Pacific Islander [52]. Comparatively, for the same school year, Black/African American students received 53.1% of the corporal punishment, disproportionately higher than that meted out to all other races. All other races (see Table 1 below) received corporal punishment less often in proportion to their racial representation in the population.

This study will further explore if these disparities influence the disproportionate administration of corporal punishment to Black American students. These statistics are important since the Mississippi Education Code allows teachers and principals to administer corporal punishment.

## 8. Methodology

Using a qualitative phenomenological research design, we interviewed 19 former students who had experienced corporal punishment in primary and secondary school in Mississippi as recently as the 2022–2023 school year. We recruited participants through both purposive and snowball sampling by contacting the participants following a study administered through Prime Panels. We interviewed participants for up to 90 min in exchange for a USD 100 Amazon gift card. Interviews were carried out via Zoom, an online virtual platform, from 21 March 2024 to 28 March 2024. For more information on how each participant self-identified in the interview, see Table 2 below. Due to the subject matter of the interviews, participants were informed prior to the interview that they could stop at any time and did not need to answer any questions that they felt uncomfortable about. Throughout the interview participants were reminded of the opportunity to stop, take a break, skip questions, reschedule, or cancel the interviews. However, even with these prompts, the researchers recognize that they could have inadvertently caused secondary trauma to participants by asking them to retell their lived experiences. This limitation could be mitigated in the future by offering participants resources to assist them with navigating the secondary trauma, providing them with coping strategies and pausing the interview at regular intervals, and offering the participants time to apply the strategies before continuing to explore their experiences [53].

Interviews were transcribed by Zoom and Rev first, and then by hand for accuracy. The process of data coding and analysis followed a three-stage framework outlined by Strauss [54].

**Figure 1:**
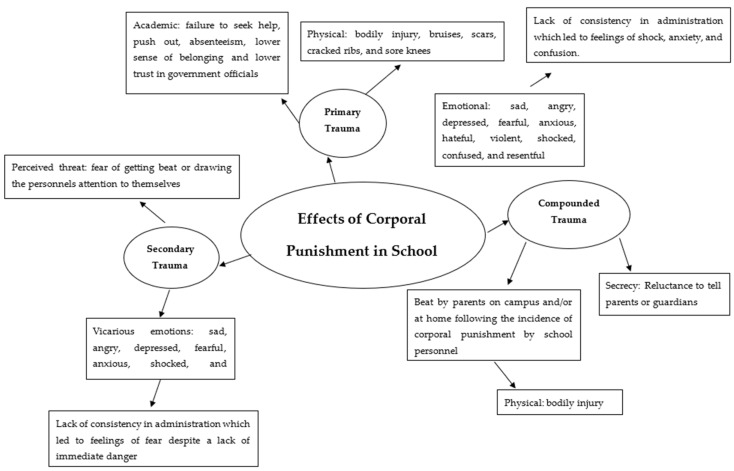
The Traumatic Effects of Corporal Punishment in Mississippi public schools. *Note:* We did not include recommendations for change, which include more government oversight and scholarly research on corporal punishment in schools as well as more humane treatment and individual therapy and counseling instead of corporal punishment in schools.

Prior to the data coding process, the transcriptions were thoroughly examined to ensure their accuracy. Coding, as defined by Silverman and Marvasti [55], is the process of categorizing data according to predetermined theoretical categories for the purpose of analysis (p. 507). Then, we performed a second cycle, thematic analysis [56,57]. To enhance the research, trustworthiness was assessed using memoing, peer debriefing, and member checking. After each interview, we recorded feelings during the research process. We recognize the importance of memoing as a tool in the process. Keeping memos after interviews was crucial, as this enabled us to explore personal emotions and shared identities, reducing bias in data collection. In addition to memoing, our team used peer debriefing to enhance in-depth reflection [58]. This analysis illuminated consistent patterns in the data, which led to our overarching themes. We found the following overarching themes: primary trauma, compounded trauma, secrecy and shame, secondary trauma, lack of consistency, racial bias, and recommendations for change (see Figure 1 for a visual display of our findings). These themes are presented in this order in the next segment of this paper.

## 9. Primary Trauma

Every participant felt pain, with most additionally feeling aggression, anger, depression, sadness, and embarrassment. For pain, each former student felt pain that lasted days and even weeks. One student, Jazz, said “I received four strokes of the cane. I remember I would never forget it was counted, and it was in class in public, so it was very, very painful. I must say it was a painful experience”. Another student recounts trying to run away from the pain of the caning: “Since the caning was very, very painful, I attempted to run, but I was held down, and to complete the strokes. So it was really, really a painful experience”. Lastly, Jim, a 25-year-old Black American man, shared that “I was in pain for weeks, you know. It affected me psychologically. You know. I just I was just so down, and the feeling was not a good one. It was not a good one”.

For aggression, some students felt aggressive toward the students and school personnel following the incident. Two students recount the aggression they felt after their corporal punishment: “it made me feel like, it made me feel like I’m fighting, fighting whoever that was paddling me there” and “I wanted to hit the lady myself”. Another common and related feeling was anger: “I was mad before, and I was mad after. I was mad”; “I felt very betrayed and and and fed up”; and lastly, “It made me mad, I was angry. I was angry and I was emotional and in pain. I was emotional because the licks hurted, they hurt”. For depression, most students felt very sad or even depressed after the incident: “I was also feeling bad and depressed because I could not feel happy again throughout my stay in school for the day. I was just so depressed because it was painful”. And “I felt so bad like, I cried, even when I was kneeling down. So it was a very bad experience, like the worst experience I had”. After having an argument with his teacher, Stanley, a 21-year-old Black American man, recounts his traumatic experience: “Well, I still have the scar on my back. Yeah, I still have the scars on my back…. before he used the ruler on me, he! He actually slapped me on the face so, and then he he used the ruler to hit my back…. he actually cracked one of my ribs”. Many former students had scars or bruises following the incident. One student recounts the depression and pain she felt following the incident: “I felt so depressed, and I felt pain all over my body because she flogged me all over my body. She even asked me to stretch out my hands”.

For embarrassment, most students felt embarrassed, often because the punishment was carried out in public in front of the class. Sammy, a 37-year-old Black American woman, recounts the embarrassment she felt: “I felt like that should have been done at least privately because now I got to worry about the classmates who aren’t my friends. Probably back there laughing. It was extremely embarrassing for me”. Likewise, Megan, a 45-year-old White American woman, recounts how the embarrassment negatively affected her academic performance: “I definitely did not learn anything after I was embarrassed”. Ultimately, these feelings and experiences were traumatic for the student—with most incidences negatively impacting their academic performance.

## 10. Compounded Trauma

Most participants received corporal punishment at home from their parents or guardians following the corporal punishment they received at school. They were also more likely to receive corporal punishment regularly in the home. This finding is supported by Taylor et al.’s [37] study, which revealed that Black Americans from southern states believe corporal punishment is a necessary component of effective parenting. One former student, Felix, an 18-year-old Black American man, recounts the following: “sometimes, they [school administration] would report me to my parent, and my dad is going to give me the beating of my life”. Moreover, most participants whose parents were available during the school day (e.g., the parents left work early or did not work) received corporal punishment first by their teacher or principal and then by their parents on the school campus. Sarah, a 31-year-old Black American woman, recounts her mother beating her at school: “So I had got a paddling that day and then my mom came up that day as well and took me in the [school] bathroom and whooped me as well”. Those parents who could not leave home beat their child once they returned home from school.

When rationalizing why his parents signed the school’s consent form that permitted corporal punishment, former student Jason, a 22-year-old Black American man, believed that it was part of the Black American culture: “Yeah, I felt like they signed it because in the Black culture, corporal punishment is even adopted in homes in the Black culture. So, I felt like they had assigned it as a way of getting me disciplined”. According to Jason, corporal punishment at home happened regularly, so it occurring at school was not unexpected or surprising. This statement of the participant is supported by the study conducted by Duong et al. [41], which indicates that Black American parents believe corporal punishment is an effective and normal method of disciplinary action that worked for them when they were children. Still, one student, who was not corporally punished at previous schools and was rarely beaten at home, felt shock; she said, “It was traumatic because like I said, I came from somewhere where that [school beatings] wasn’t an option. I didn’t know anything about it. It wasn’t nothing that I was aware of it. Well, I’m not going to say kind of, it scarred me”. Most students experienced compounded trauma at home and on campus which, in turn, led to subsequent secrecy and shame.

## 11. Secrecy and Shame

Students who experienced corporal punishment at school often did not tell their parents for fear of additional disciplining at home. Laila, a 22-year-old Black American woman, talks about having to keep this traumatic experience a secret: “I couldn’t tell my parents about it, because I know they wouldn’t stand with me. They might give me another punishment”. In contrast, Tony, a 24-year-old Black American man, said he told his father, who just laughed at him. His father told him not to take the incident seriously and that students are always flogged in school. CPS is normal. As a result of his father laughing at him, he did not tell anyone else about the harsh beating. He felt like he was solely responsible for the traumatic experience. Mindy, a 23-year-old Black American woman, had a similar experience: “At that time [during elementary school], I wasn’t with my parents, so I was staying with my elder sister, I told her, but she just laughed over it. She said: That’s how they do, they flog students in school when they misbehave. So, she advised me not to do anything that leads to them flogging me again, not to go against the school rules and regulations”. CPS lead to primary and compounded trauma as well as secondary trauma.

## 12. School Culture and Peer Effects

The only consistent thing about corporal punishment in Mississippi was how often it was administrated to students and how it negatively impacted the students who received the corporal punishment and their peers who did not. One student recounts that a peer was punished for stealing something: “He was flogged severely. They flogged him mercilessly. He had wounds, he sustained some wounds on his body… I was scared”. Another student, 21-year-old Jamal, recounts a traumatic experience for him when his friend was beaten for something he did not do: “He had a marks on his back, and he cried. I actually helped him to the school clinic. He actually needed treatment. And then his dad came and picked him up. And that was the last I saw of him… I felt angry because he [the teacher] was actually punishing the wrong person, and it was not really that necessary to actually beat him”. Other students were punished for making noise/talking, missing or performing poorly on assignments, tardiness, not being where they were supposed to be at a certain time, etc. Another student, Rally, a 21-year-old Black American man, believes that “corporal punishment is like the norm, or like a culture being beaten by the Mississippi personnel”. Corporal punishment was part of the school culture and often led to secondary trauma experienced by peers and friends. There is a significant impact of secondary trauma on a child’s cognitive ability, their emotional health, their behavior, their physical health, and their personal relationships [27]. Students did not know when to expect CPS for themselves or their peers, which resulted in more trauma.

## 13. Lack of Consistency

There was a general lack of consistency in how corporal punishment was administered, with incidences ranging from being slapped in the face, made to crawl across pavement, or hold painful positions for long periods of time. Perpetrators were usually teachers and principals, but sometimes even other students, mainly high school seniors, which is a violation of a school law that states that only teachers, assistant teachers, principals, and assistant principals can administer corporal punishment in Mississippi. This inconsistency aligns with the findings of Smith and Hains [46], who emphasize that school discipline policies vary in accordance with the philosophies and beliefs of educators guiding discipline processes. Perpetrators hit students with their hands, paddles, or canes on the buttocks and thighs. Some students were also slapped in the face, pinched, or made to hold painful positions for long periods of time. For example, Mindy, a Black American woman, recounts: “We were asked to kneel down and carry up a heavy load in our hands, and after sometimes when the teacher when we were getting exhausted, she would then flog us on the back” with a cane. Some students were even asked to crawl back and forth across the floor, which hurt.

## 14. Racial Biases

There were certain distinctions between White American and Black American participants, with Black American participants often receiving CPS while their White American peers were exempt. For example, Tony, a Black American man who was punished after getting into a heated argument with a fellow student who was a White American, said: “The teacher flogged me, and also asked me to kneel down for a very long time, but the White students was not punished”. He believed the incident was racist and that the teacher, who was Black, was operating out of fear: “I felt she was working on the instructions of a White teacher. I felt maybe she was scared of what will happen if she flogged the White student”. When talking about how he was paddled seven or eight times for a fight he had with a White American boy, Jim remembers that “The white boy was not punished…I can’t even, you know, face on the facts that I was beaten. And yeah, I stayed away from the school for like about three days or so before I continued”. Black American students often felt that they were being targeted and treated as less than human because they were Black American by both White American and Black American school personnel and students. Overall, former Black American students felt that White American students who committed the same infraction were not punished.

## 15. Strained Relationships and Push-Out

Following the corporal punishment, students felt dislike or hatred for their teachers, principals, and students. Additionally, they were more likely to skip school or drop out following the incident. Bella, a 25-year-old woman, recounts the strained relationship she experienced with her teacher after she was caned repeatedly all over her body: “It can also lead to emotional distress like fear and anxiety. I couldn’t approach her. So that was how it affected my academic performance, because I was always scared of her”. Another former student, a 24-year-old Black American man, similarly feels that the incident “really affects my relationship [with the teachers and principal] because most times, if I have any problem, I wish I would have talked to them, but I wasn’t able to talk to them because I was actually scared”. Other students hated their teacher or principal for inflicting pain on them. Two students recounted that they hated their teachers: “I almost hated the teacher” and “it made me hate that teacher at that moment. I hated her so much for inflicting that bruise on me and I wished that it wasn’t that way”. Other former students stayed away from school following the incident, while other former students wanted to change schools or drop out.

## 16. Recommendations for Change

Students had useful insights into recommendations for change, such as more government oversight and scholarly research on CPS as well as more humane treatment and individual therapy and counseling instead of CPS. For example, Jamal, a Black American man, argues that school personnel should recognize the full humanity of their students: “if we are being treated as human, treated as part of the community, yeah, I feel we would actually get along better”. Stanley, a 21-year-old Black American man, argues that “it should actually be something the government should look into, because most schools actually misuse corporal punishment for their own benefit, you know. Yeah, like a teacher because he has authority over the student, he would want to punish the kids for his own pleasure”. He felt like that was the case for him and his friends. Moreover, Tony believes that “the government should try to oversee what is happening in schools. Try to, you know, try to minimize the rates of corporal punishment in school so that this will not affect the students”. This suggestion aligns with the findings of Irby and Clough [43], who highlighted that it is essential that teachers practice consistency in order to ensure that their students are treated fairly and equally. Moreover, Jason believes that more research is needed to ensure that corporal punishment is the best option and whether something else will work better. Lastly, Rally, a Black American man, said that he felt trained professionals (e.g., psychologists and counselors) were needed to address the underlying root causes of student misbehavior. Overall, former students had unique insights into recommendations for change, having experienced corporal punishment and witnessed it among peers.

## 17. Discussion

We found the following overarching themes: (1) students experienced primary trauma at school following CPS and compounded trauma when their parents found out about their misbehavior; (2) students often did not tell their parents about their punishment for fear of an additional punishment, and instead shouldered the burden alone; (3) there was a lack of consistency in how corporal punishment was administered; (4) corporal punishment was part of the school culture and negatively affected peers, leading to secondary trauma; (5) many Black American participants felt that there were racial biases at play wherein their White American peers were exempt from punishment, but they were not for committing the same infractions; and (6) former students had multiple recommendations for change. The most common were more academic research, government oversight, and individual counseling for the misbehaving student over corporal punishment, specifically, more research on corporal punishment and disparities and more government oversight of the execution of corporal punishment.

These findings align with and extend prior findings. For example, Cruz et al. [9] found that students who experienced physical trauma felt less faith in their perpetrators and in authority figures more generally. Likewise, our participants felt less trust in school officials, parents, and guardians due to corporal punishment. Additionally, the American Psychiatric Association studied the psychological consequences of traumatic exposure and found that youth who experienced trauma had post-traumatic stress [12]. Likewise, our participants felt fear, anxiety, and depression following their instance(s) of trauma. Lastly, Benight and Bandura [29] found that following a traumatic incident, youth had an inability to recover. Likewise, former students often recounted how the event(s) scarred them for life—how they will never forget their experiences with corporal punishment. It is imperative that children be provided with a safe, nonthreatening environment in which safety and the establishment of trust are prioritized in order to heal trauma such as secondary trauma. They also felt that the instances affected their current behavior as adults.

Though our findings align well with the previous literature, we also make valuable contributions to it. First, students experienced compounded trauma on campus by both parents and teachers, and at home by parents and guardians. While prior research finds that students who experience corporal punishment at home are more likely to experience it at school, prior research has yet to find how students are beaten on campus and off-campus by their parents or guardians following and related to incidents of corporal punishment. In that context, students felt fear that if they told their parents, they would be beaten twice—once at school and once at home. As such, students often kept their instances of corporal punishment at school a secret.

More research is needed on the administration and effects of CPS: for example, “corporal punishment in public schools persists in over a third of U.S. states, yet very little is known about how it is administered or what potential unintended effects it may have on students” [34], p. 8. We found that there was a general lack of consistency in how punishment was administered and who was allowed to administer it. While research has previously documented physical assault, there is limited information on students’ experiences with painful positions (sitting on the wall) or tasks (crawling on gravel). These instances of corporal punishment reflect a keen form of school torture that should be further explored in the literature. Moreover, while research has well documented the level of trauma felt by victims who receive corporal punishment at school, there is an acute gap in research on the peer effects of trauma. Overall, secondary trauma among children is limited, but it is important to fully theorize the phenomenon of childhood trauma. We found that students experienced trauma when they saw their friends or classmates being beaten regardless of whether they themselves were in immediate danger.

Moreover, while research has quantified racial disparities particularly between White American and Black American students, there is a lack of research on the racial demographics of the perpetrator. Here, we find that both Black American and White American teachers and principals are guilty of beating Black American children for the same infractions that White American children are not punished for and how that negatively impacted the punished students’ participation in school and psychological well-being. Lastly, we share recommendations for change from those who have experienced corporal punishment. While research aligns well with these recommendations, it is critical to give voice and space to those who were traumatized to share recommendations for change.

Although not discussed explicitly, we also found that participants experienced a lack of trust in the government (e.g., a reluctance to speak to school personnel or ask for help) and in their parents and guardians (e.g., a tendency to keep the CPS a secret). While research aligns with this finding, we extend it to include the parents (e.g., not just the school personnel). These findings are important to understanding the full scope of trauma experienced by students in Mississippi, which we believe generalizes to the other 22 states that allow corporal punishment. Our last contribution and arguably the most important is this: corporal punishment is still happening in American schools. We hope that this contemporary analysis of corporal punishment practices in American schools published in a flagship journal will remind the world of the legally state-sanctioned violence happening every day in American schools—a practice that is antithetical to American ideals and, we believe, violates our schoolchildren’s fundamental human rights.

In terms of implications, we call for the abolishment of CPS. We ask educational stakeholders to push for the reversal of CPS legality because it is ineffective and traumatic. At the very least, students and families should be allowed due process. CPS takes an emotional and psychological toll on students, which demonstrates the need for comprehensive support systems in schools. There is a lack of information and resources available to teachers to address trauma in their daily practice [59]. Additionally, the measurement of secondary trauma has received relatively little research attention, and most of the assessment devices available are designed for therapists rather than for the general public or for children [26]. To facilitate the development of trauma-informed instructional practices, educators will need to gain a deeper understanding of the implications that complex trauma can have for brain development [60]. A teacher or teaching assistant with appropriate training can achieve results similar to those provided by a trained therapist [61].

Implications of the study call for concerted efforts from educators, policymakers, and parents to prioritize the well-being and dignity of all students. Addressing fear, eliminating gender and racial disparities, providing support for affected children, and promoting parental engagement will create safer and more nurturing learning environments for every child in US schools. Schools should not use corporal punishment as a disciplinary strategy because it has negative psychological effects, is ineffective, poses a risk of physical injury, and negatively impacts students’ academic performance. Rather, educators should implement nonviolent disciplinary methods that promote positive behavior, respect children’s rights, and ensure a safe and supportive learning environment. Again, so long as corporal punishment is used in schools, students should have an opportunity for due process—something they currently do not have.

## 18. Conclusions

This study makes an important contribution to our understanding of how former victims of corporal punishment in schools process traumatic events. The findings fill a gap in the literature related to corporal punishment in schools and help us understand the critical role school personnel, parents, and even students play in shaping the academic outcomes, shock, and psychological stress students face when exposed to corporal punishment regularly. This study also demonstrates the value of applying childhood trauma theories to K-12 research, in this case, by focusing on archaic educational structures and practices as problematic rather than placing all blame on misbehaving children. Future research on the topic of corporal punishment in American schools should deepen and extend the use of childhood trauma as a conceptual framework to guide study design, research methods, and analysis. Our capacity to create positive, instead of traumatic, experiences for students is dependent on our ability to think in new ways about old laws like corporal punishment in schools.

## Figures and Tables

**Table 1:** National data on population and instances of corporal punishment by racial group.

	Black/African American	White American	Hispanic/Latin *	Two or More Races	Asian	American Indian/Alaskan Native	Native Hawaiian/Pacific Islander
Enrollment	47.74%	43.13%	4.39%	3.33%	1.15%	0.22%	0.06%
Corporal Punishment	53.1%	43.0%	1.9%	1.2%	<1.0%	<1.0%	0%
Teachers	22.6%	75.6%					
Principals	37.1%	62.9%					

[52]. The asterisk is used to indicate both genders or (Latin(a/o) and not to indicate a need for a notation.

**Table 2:** Self-identified race, gender, and age of each participant along with their pseudonym.

Number	Name	Race	Gender	Age
1	Mark	Black American	Man	20
2	Rally	Black American	Man	21
3	Bella	Black American	Woman	25
4	LaShawna	Black American	Woman	38
5	Laila	Black American	Woman	22
6	Tony	Black American	Man	24
7	Jim	Black American	Man	25
8	Jason	Black American	Man	22
9	Jamal	Black American	Man	21
10	Felix	Black American	Man	18
11	Jazz	Black American	Man	20
12	Stanley	Black American	Man	21
13	Sammy	Black American	Woman	37
14	Mindy	Black American	Woman	23
15	Sarah	Black American	Woman	31
16	Christina	Black American	Woman	49
17	Adam	White American and American Indian	Man	58
18	Megan	White American	Woman	45
19	Janet	White American	Woman	53

## Data Availability

The raw data supporting the conclusions of this article will be made available by the authors on request due to privacy of the participants.
